# Differences in TCR-Vβ Repertoire and Effector Phenotype between Tumor Infiltrating Lymphocytes and Peripheral Blood Lymphocytes Increase with Age

**DOI:** 10.1371/journal.pone.0102327

**Published:** 2014-07-14

**Authors:** Hongwei Shao, Yusheng Ou, Teng Wang, Han Shen, Fenglin Wu, Wenfeng Zhang, Changli Tao, Yin Yuan, Huaben Bo, Hui Wang, Shulin Huang

**Affiliations:** 1 Guangdong Province Key Laboratory for Biotechnology Drug Candidates, Guangzhou, China; 2 School of Biosciences and Biopharmaceutics, Guangdong Pharmaceutical University, Guangzhou, China; 3 Southern Medical University, Guangzhou, China; Université Libre de Bruxelles, Belgium

## Abstract

Tumor infiltrating lymphocytes (TIL) reflect the host's anti-tumor immune response, and can be a valuable predictor of prognosis. However, many properties of TIL are not fully understood. In the present study, TCR-Vβ repertoires of cancer patients were primarily analyzed by flow cytometry. Abnormally expressed TCR-Vβ subfamilies were generally found in both TIL and peripheral blood lymphocytes (PBL) of each patient. Of note, increased patient age was associated with increasingly biased TCR-Vβ repertoire in TIL but not in PBL, and the dispersion degree of the differences of TCR-Vβ subfamilies between TIL and PBL correlated positively with age (*P* = 0.007). Utilizing immunoscope analysis, we identified the age-related reduction in TCR-Vβ diversity, but polyclonal pattern was predominant in significantly expanded TCR-Vβ subfamilies. In addition, we found that older patients possessed a decreased ratio of CD8^+^CD62L^+^ non-effector cells in TIL compared to PBL, implying age-related increase of CD8^+^CD62L^−^ effector cells in TIL. The colocalization analysis of CD8 and CD3, however, suggested the suppressed activity of these effector cells in tumor microenvironment. These findings further elucidate the properties of TIL, showing an increasing difference between TIL and PBL with age, which may provide insight for the development of effective immunotherapies for cancer patients of different ages.

## Introduction

The cellular components of the immune system play an important role in tumor initiation and development. Burnet and Thomas speculated several decades ago that lymphocytes act as sentinels in recognizing and eliminating continuously arising, nascently transformed cells [1]. Now, it is well known that cancers in general elicit cellular immune responses, and T cells directed against tumor-associated antigens (TAA) are frequently detected in cancer patients [2].

Tumor infiltrating lymphocytes (TIL) are thought to contain tumor-specific cells that reflect tumor-host interactions [3]–[5]. The presence of TIL seems to correlate with a favorable prognosis in some tumor types [6]–[8]. However, in patients with advanced stages of cancer, TIL are functionally impaired and unable to prevent progressive tumor outgrowth [9]–[11]. Despite being impacted by the tumor microenvironment, TIL can easily be expanded and activated *in vitro* [12]. Such *in vitro* activated TIL have been used in autologous adoptive T cell therapy/transfer (ACT) to yield durable and complete responses in subpopulations of cancer patients [13]–[15]. Thus, characterization and tracking of the T-cell receptors (TCR) utilized by TIL may reveal important information about the biology of anti-tumor T cell responses [16].

Many studies have demonstrated a skewed TCR repertoire within TIL compared to peripheral blood lymphocytes (PBL) [17]–[19]. By cloning of TCR sequences followed by sequencing, it was shown that clonal expansions with restricted TCR beta and alpha chain variable regions (TCR-Vβ and TCR-Vα) were common within TIL in most of the tumors studied [20], [21]. The sequences of clonally expanded T cells indicated that TAA-induced immune responses led to the proliferation of specific subsets of TIL. Most studies have focused on the expanded TCR subfamilies themselves, with or without focusing on MHC restriction [17]. However, few studies have focused on the regular pattern of TCR expression difference between TIL and PBL. To better understand the ongoing immune response to tumors and develop effective immunotherapies, it is important to explore the differences in TCR repertoire between TIL and PBL of the same patient.

In the present study, the TCR-Vβ gene repertoire of PBL and TIL from individual patients was analyzed by flow cytometry (FCM) and complementarity determining region 3 (CDR3) length distribution pattern. The results showed that the dispersion degree of the differences of TCR-Vβ subfamilies between TIL and PBL correlated positively with age. Although there was an age-related reduction in TCR diversity within TIL, a polyclonal pattern was predominant in significantly expanded TCR-Vβ subfamilies. In addition, phenotypic analysis indicated that while CD45RO^+^CD8^+^ cells showed no clear trend, the ratio of CD8^+^ CD62L^+^ non-effector cells in TIL compared to that in PBL correlated negatively with age, which implied age-related increase of CD8^+^CD62L^−^ effector cells in TIL. The colocalization analysis of CD8 and CD3, however, suggested that the functional activity of these effector cells were suppressed in tumor microenvironment.

## Materials and Methods

### Patients and controls

Eleven patients (3 female and 8 male; 5 with lung cancer, 4 with colon cancer, and 2 with liver cancer) with a mean age of 52 years (ranging from 32–71), admitted to Dongguan City People's Hospital, were included in this study (Table 1). Five healthy controls (1 female and 4 male) whose mean age was 50 years (ranging from 33–65) were also included. All patients signed the informed consent document. Healthy donor blood samples were taken from volunteers who signed an informed consent document. The protocols used for human studies were approved by the Medical Ethics Committee of the Dongguan City People's Hospital and GDPU.

**Table 1:** Cancer patients.

Patient	Cancer location	Age	Gender	Stage
PL1	lung	35	male	IV
PL2	lung	59	male	III
PL3	lung	63	female	IV
PL4	lung	65	male	IV
PL5	lung	67	male	IV
PC1	colon	38	male	IV
PC2	colon	44	female	IV
PC3	colon	58	male	III
PC4	colon	71	male	IV
PH1	liver	32	male	IV
PH2	liver	42	female	IV

PL, Patient with lung cancer; PC, Patient with colon cancer; PH, Patient with liver cancer.

### Isolation of TIL

Referring to previous reports [11], [22], [23], ascites or pleural effusion of patients were freshly harvested and centrifuged at 800×g for 10 min at 18–20°C. The supernatants were discarded, the cells were suspended in PBS, and lymphocytes were isolated with Ficoll-Paque™ PLUS (GE Healthcare, Sweden) according to manufacturer's protocol. Isolated TIL were suspended in RPMI 1640 medium with 10% fetal bovine serum (FBS).

### Collection of peripheral blood specimens

Peripheral blood specimens were collected by venipuncture with a vacuum blood collection tube containing EDTA-K2. Collected specimens were stored at 4°C (no more than 8 hours) for further processing.

### Flow Cytometry (FCM)

The TCR-Vβ repertoire was determined by four-color flow cytometry with TCR Vβ Repertoire Kit (Beckman Coulter, Marseille, France), which consists of a set of monoclonal antibodies (mAb) designed to label 24 distinct human TCR-Vβ subfamilies. In this kit, the 24 TCR-Vβ antibodies are divided into 8 groups. Each group includes three distinct TCR-Vβ antibodies labeled with phycoerythrin (PE), fluorescein isothiocyanate (FITC), or PE plus FITC. The nomenclature used for Vβ subfamilies is the same as that used by Wei et al. [24].

For immunostaining, 20 µl of each group of TCR-Vβ antibodies and 10 µl of CD3 antibody labeled with Phycoerythrin Cyanin 5.1 (PE-Cy5) (for gating of CD3^+^ lymphocytes) were mixed with 100 µl of TIL (5–10×10^5^ cells) or peripheral blood, and incubated at room temperature for 20 minutes in the dark. Then erythrocytes were lysed, washed, and fixed according to the recommended protocol. Data acquisition and analysis were performed on an EPICS XL flow cytometer using EXPO32™ ADC software (Beckman Coulter, Fullerton, USA).

### Evaluation of TCR-Vβ expression difference between TIL and PBL

To analyze the expression difference of TCR-Vβ repertoire between TIL and PBL, Δ values were calculated as the percentage of each TCR-Vβ subfamily in CD3^+^ TIL minus the counterpart in CD3^+^ PBL. To evaluate the dispersion degree of expression differences of TCR-Vβ subfamilies between TIL and PBL, the variance of Δ values of 24 TCR-Vβ subfamilies was subsequently computed for each patient.

### RNA extraction

Total RNA was extracted from 5–10×10^6^ cells using TRIzol reagent (Invitrogen, Carlsbad, USA), and the purity and concentration were determined by the absorbance at 260 nm and 280 nm.

### cDNA synthesis and CDR3 length polymorphism analysis of TCR-Vβ

With primer specific for constant region of TCRB (TCRBC) (5′-CTCAAACACAGCGACCTC), the first strand cDNA synthesis was performed using PrimeScript II 1st Strand cDNA Synthesis Kit (Takara, Japan) according to manufacturer's protocol. Based on Akatsuka et al's work [25], a set of primers for the amplification of CDR3-containing fragment of TCRB were designed for applying to GenomeLab™ GeXP Genetic Analysis System (Table S1). Multiplex PCR was performed with these mixed primers (universal primers: specific primers = 60∶1 mole ratio). After cleaning, the amplified products were subjected to capillary electrophoresis with GenomeLab™ GeXP Genetic Analysis System, and data were analyzed using GenomeLab eXpress Profiler (Beckman Coulter, Fullerton, USA).

### T cell phenotypic analysis

With different combinations of the following directly conjugated mouse mAbs against human antigens (CD8-FITC, CD3-PE-Cy5, CD62L-PE, and CD45RO-ECD), PBL and TIL were stained as previously described [26]. All mAbs were from Immunotech (Beckman Coulter, Marseille, France). Data acquisition and analysis was performed on an EPICS XL flow cytometer using EXPO32™ ADC software (Beckman Coulter, Fullerton, USA).

### Colocalization analysis of CD3 and CD8 molecules in T lymphocytes

TIL and PBL from healthy controls, either freshly isolated or stimulated with 1000 U/ml IFN-γ added on day 0, 50 ng/ml OKT3 added on day 1, and 500 U/ml IL-2 included in the medium from day 1 onward to the end (day 6), were separately incubated with mAbs (CD8-FITC and CD3-PE-Cy5) for 30 min in dark. After washing with PBS and fixing with 2% paraformaldehyde, the cells were observed with confocal laser scanning microscope Fluoview FV1000 (Olympus, Tokyo, Japan). The colocalization of CD3 and CD8 was analyzed, and colocalization index (r) was calculated by FV10-ASW2.1 software with the equation:
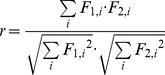

*F*_1,*i*_: Brightness of wavelength *λ*_1_ at *i*-th pixel; *F*_2,*i*_: Brightness of wavelength *λ*_2_ at *i*-th pixel.

### Statistical analysis

SPSS software v.19.0 (IBM, Armonk, USA) was used to calculate the correlation coefficient and statistical significance between variances of TCR-Vβ expression Δ values and patients' age. Spearman's rank-order correlation was adopted, and P values less than 0.05 were considered to be statistically significant. Statistical significance for colocalization index (r) of CD3 and CD8 was evaluated with Student's *t* test using the data analysis tool of Microsoft Excel software with P less than 0.05 as the criterion of significance.

## Results

### Changed TCR-Vβ expression in TIL and PBL of cancer patients

**Figure 1:**
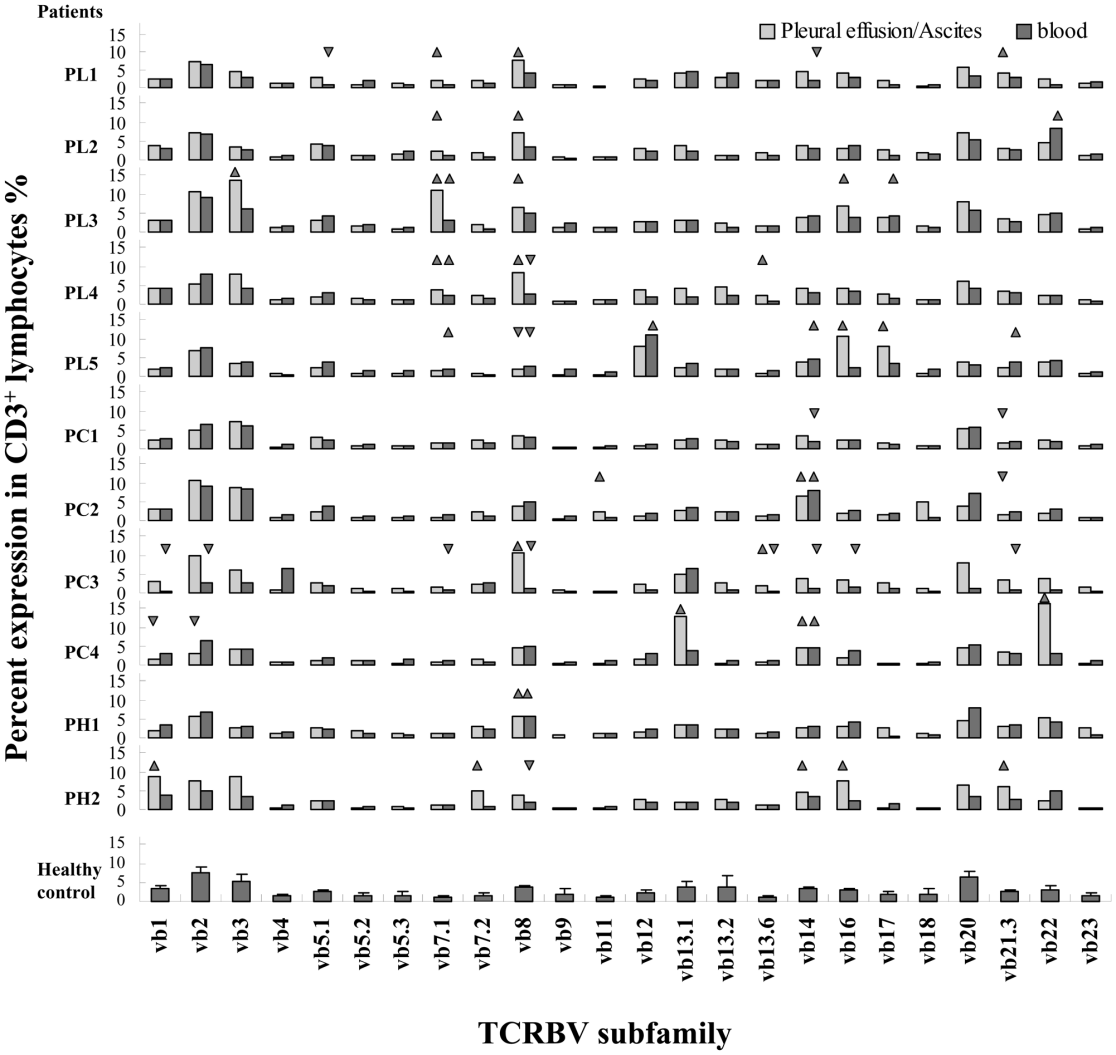
TCR repertoire in TIL and PBL of cancer patients. The TIL or peripheral blood lymphocytes were stained with anti-CD3 and 24 anti-TCRBV antibodies from a TCR Vβ Repertoire Kit. After lysing erythrocytes, washing, and fixing, TIL or PBL were subjected to FCM analysis. For a given TCRBV subfamily expression, any value higher or lower than the mean of the healthy control group, plus or minus 3 SD, was considered abnormal expression (higher labeled with triangle ▴; lower labeled with inverted-triangle ▾). PL indicates patient with lung cancer, PC indicates patient with colon cancer, PH indicates patient with liver cancer.

A skewed TCR-Vβ repertoire in TIL and PBL of cancer patients compared to age-matched healthy controls was found (Fig. 1). For a given TCR-Vβ subfamily expression level, any value outside of the mean of the healthy control group, plus or minus 3 SD, was considered abnormal [27]. Abnormal expression was found in all eleven patients.

Although Vβ8 expression levels were increased in six TIL specimens, overexpression of Vβ8 was most obvious in TIL isolated from lung cancer patients (four out of five). Reduced Vβ8 expression was found in four PBL specimens. Vβ7.1 was increased in four TIL and three PBL specimens derived from five patients. Vβ14, Vβ16, Vβ21.3, Vβ13.6, Vβ17, and Vβ22 were overexpressed in more than one patient. In addition, overexpression of six other Vβ subfamilies was identified (Fig. 1).

Including Vβ8, nine Vβ subfamilies were reduced in patient specimens. In some cases, reverse expression levels of TCR-Vβ subfamilies were observed between TIL and PBL from the same patient; for example, Vβ8 in patient PL4 and PC3, and Vβ13.6 in patient PC3. These expression changes, downregulated in PBL and upregulated in TIL, imply an oriented T cell migration and subsequent activated immune response.

### Age-related differences in TCR-Vβ repertoire between TIL and PBL

**Figure 2:**
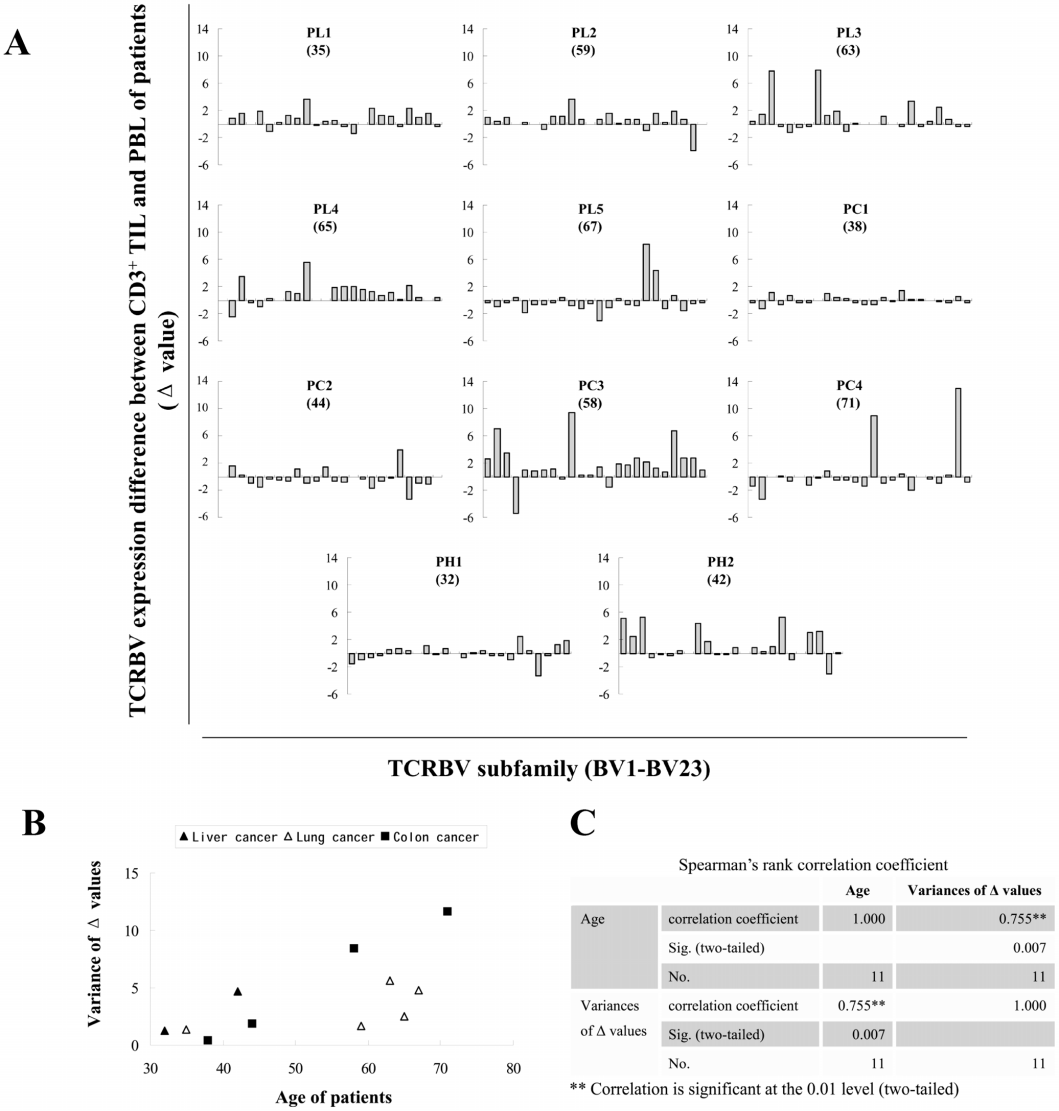
Analysis of expression difference of TCR-Vβ repertoire between TIL and PBL of cancer patients. (**A**) Expression difference of TCR-Vβ subfamily between TIL and PBL (Δ value) is calculated as the percentage of each TCR-Vβ subfamily in CD3^+^ TIL minus the counterpart in CD3^+^ PBL. All 24 Δ values (for 24 TCR-Vβ subfamilies) were used to generate a histogram for each patient. The numbers in brackets are the ages of patients. (**B**) The dispersion degree of Δ values is expressed as variance. The variances were subsequently matched with the age of the patient to generate a scatter plot. (**C**) The linear correlation coefficient between variance and age was subsequently computed by SPSS software with Spearman's rank-order correlation model.

To quantify the difference in TCR-Vβ expression between TIL and PBL of the same patient, the Δ values (the percentage of each TCR-Vβ subfamily in CD3^+^ TIL minus the counterpart in CD3^+^ PBL) were calculated and shown in Figure 2A. The dispersion degree of Δ values from different TCR-Vβ subfamilies vary obviously from patient to patient.

To evaluate the dispersion degree of Δ values, the variance of the Δ values was calculated. The variances were subsequently matched with the age of the patient to generate a scatter plot (Fig. 2B). Interestingly, there was a positive linear correlation trend between variance and age. To compute the linear correlation coefficient, we utilized SPSS software, and Spearman's rank-order correlation model was selected. With a P value of 0.007 and correlation coefficient of 0.755, the data showed a highly significant positive correlation between Δ value variance and age (Fig. 2C). This result indicates that increased age is associated with an elevated dispersion degree of TCR-Vβ expression differences between TIL and PBL in cancer patients.

### Differences were caused by changed TCR-Vβ expression in TIL not in PBL

Increased dispersion degree of TCR-Vβ expression differences between TIL and PBL with age may be caused by markedly altered expression of TCR-Vβ subfamilies in TIL, in PBL, or in both. To examine these possibilities, the expression level of each TCR-Vβ subfamily in PBL or TIL from each patient was separately compared with the mean of the counterparts in PBL of healthy controls.

**Figure 3:**
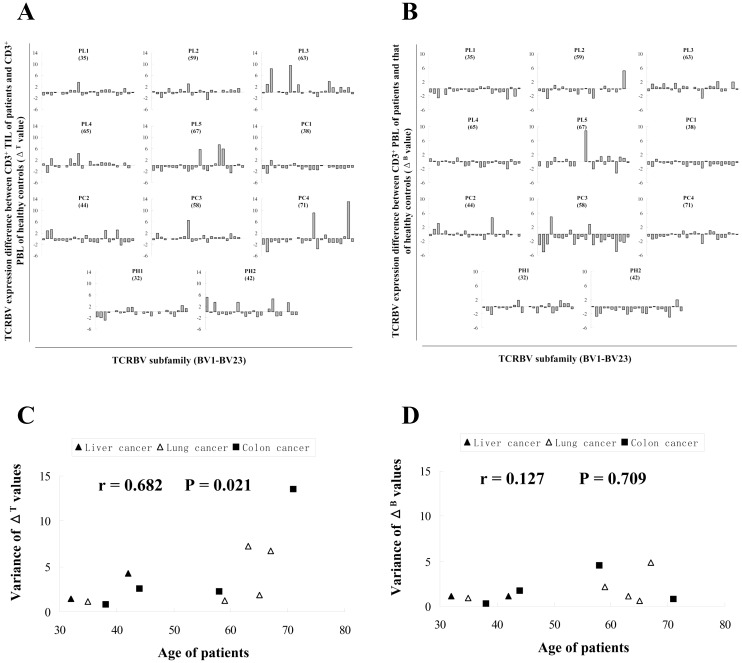
Analysis of expression difference of TCR-Vβ repertoire between TIL or PBL of cancer patients and PBL of healthy controls. The expression level of each TCRBV subfamily in PBL or TIL of each patient was separately compared with the mean of the counterparts in PBL of healthy controls. (**A**) Δ^T^ values (the percentage of each TCR-Vβ subfamily in CD3^+^ TIL of patient minus the mean percentage of the counterpart in CD3^+^ PBL of healthy controls) were calculated to generate the histograms. (**B**) The variances of Δ^T^ values for each patient were calculated and matched with the ages, and the correlation coefficient (r) and P value (P) were computed. (**C**) Δ^B^ values (the percentage of each TCR-Vβ subfamily in CD3^+^ PBL of patient minus the mean percentage of the counterpart in CD3^+^ PBL of healthy controls) were calculated to generate the histograms. (**D**) The variances of Δ^B^ values for each patient were calculated and matched with the ages, and the correlation coefficient and P value were subsequently computed. The numbers in brackets are the ages of patients.

As described above, the Δ^T^ values (the percentage of each TCR-Vβ subfamily in CD3^+^ TIL of the patient minus the mean percentage of the counterpart in CD3^+^ PBL of healthy controls) were calculated (Fig. 3A). The variances of Δ^T^ values for each patient and the correlation coefficient were subsequently computed (Fig. 3B). With a correlation coefficient of 0.682 and P value of 0.021, a significantly increased fluctuation of TCR-Vβ expression in TIL of older patients was confirmed.

Likewise, Δ^B^ values (the percentage of each TCR-Vβ subfamily in CD3^+^ PBL of the patient minus the mean percentage of the counterpart in CD3^+^ PBL of healthy controls) were also calculated (Fig. 3C). The variances of Δ^B^ values for each patient and the correlation coefficient were similarly computed (Fig. 3D). A correlation coefficient of 0.127 and P value of 0.709 indicated that, for PBL specimens, there was no significant correlation between TCR-Vβ expression fluctuation and patient age.

### Decreased TCR-Vβ diversity in TIL with age and predominant polyclonal pattern

**Figure 4:**
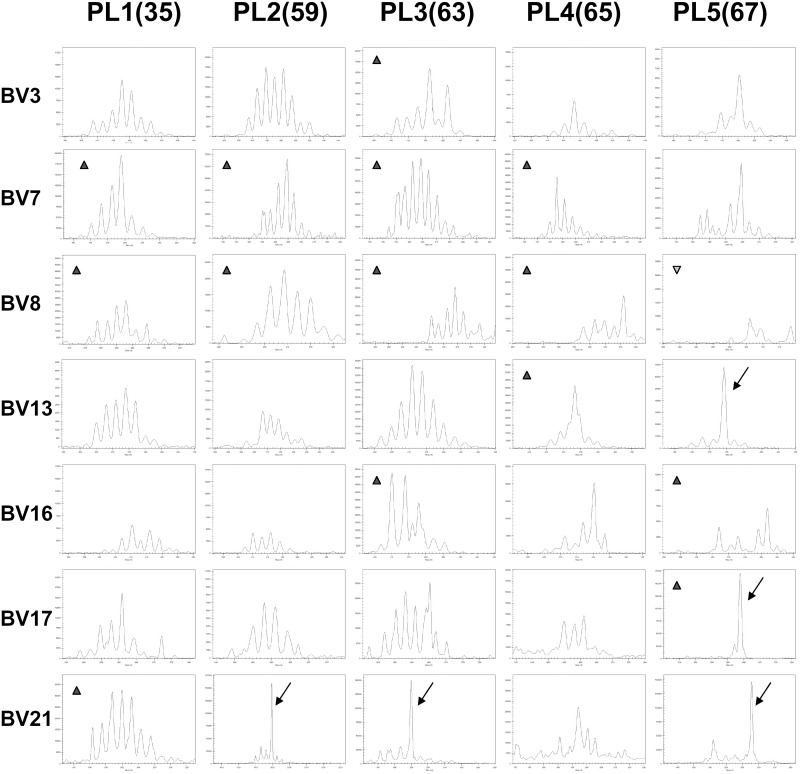
Analysis of clonal expansion in TIL of 5 lung cancer patients. Seven TCR subfamilies (BV3, BV7, BV8, BV13, BV16, BV17, and BV21) overexpressed in TIL of five lung cancer patients were selected to show the CDR length distribution. Decreased numbers and/or biased distribution of CDR3 peaks with increased age of patients were shown. TCRBV21, which did not display remarkable overexpression by FCM detection, but showed oligoclonal/monoclonal expansion pattern in 3 out of 5 TIL specimens, was selected as the control. The numbers in brackets are the ages of patients. The arrows point to monoclonal expansion peaks. The triangle (▴) means overexpression, and inverted-triangle (▾) means underexpression, as shown in Figure 1.

To explore the clonal expansion pattern in TIL, five specimens from lung cancer patients were selected for immunoscope (CDR3 length polymorphism) analysis, and seven TCR subfamilies (BV3, BV7, BV8, BV13, BV16, BV17, and BV21) overexpressed in TIL of lung cancer patients were selected to show the CDR length distribution (Fig. 4). It was found that increased age of patients was related to the decreased numbers and/or biased distribution of CDR3 peaks overall, which suggested decreased TCR-Vβ diversity in TIL with age.

Although oligoclonal/monoclonal expansion patterns were found in TIL specimens, the patterns did not correspond with high overexpression levels of TCR-Vβ subfamilies. We found that Vβ8 was overexpressed in four out of five lung cancer patients (Fig. 1), however, there was no TIL specimen in which Vβ8 showed a monoclonal expansion pattern (Fig. 4). Vβ7.1, another overexpressed subfamily in TIL, showed no monoclonal expansion pattern in all five TIL specimens. Indeed, Vβ17 from patient PL5 was the only subfamily that displayed a significantly increased expression level and a simultaneous monoclonal expansion pattern. Interestingly, TCR-Vβ21, which did not display remarkable overexpression by FCM detection, did display an oligoclonal/monoclonal expansion pattern in 3 out of 5 TIL specimens (Fig. 4).

Immunoscope analysis indicated that, although oligoclonal/monoclonal expansion patterns appeared to increase with age, many TCR-Vβ subfamilies overexpressed in TIL specimens showed a polyclonal expansion pattern.

### Decreased ratio of CD8+CD62L+ cells in TIL to that in PBL with age

**Figure 5:**
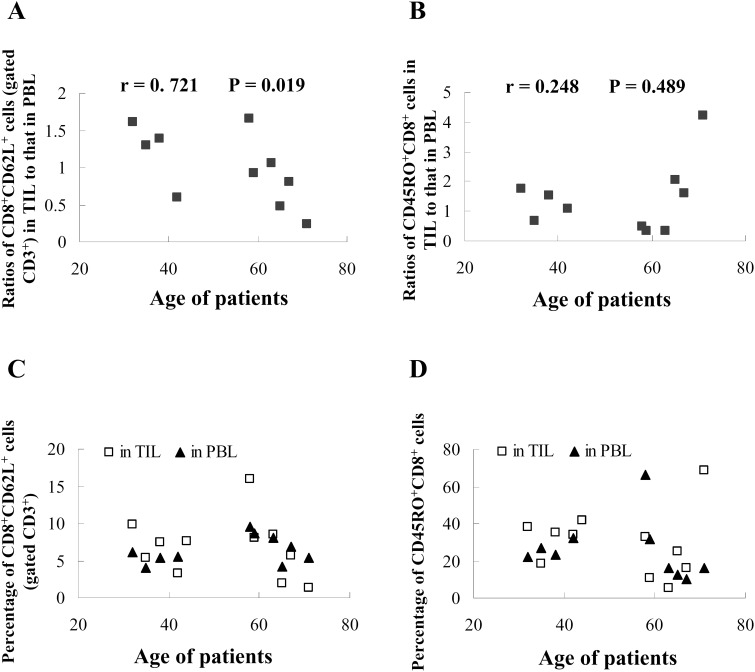
Changed T cell subsets. The freshly isolated TIL or peripheral blood was stained with directly conjugated mouse mAbs against human antigens, CD45RO-ECD/CD8-FITC, or CD3-PC5/CD8-FITC/CD62L-PE. The ratios of percentage of CD8^+^CD62L^+^ subset (gated CD3^+^ cells) in TIL to that in coupled PBL (**A**) and percentage of CD45RO^+^CD8^+^ subset in TIL to that in coupled PBL (**B**) were matched with the ages of patients. The coupled percentages of CD8^+^CD62L^+^ subset (gated CD3^+^ cells) in TIL and PBL (**C**) and CD45RO^+^CD8^+^ subset in TIL and PBL (**D**) were matched with the ages of patients. The data for PBL of patient PC2 (age 44) is not available.

The loss of surface L-selectin (CD62L) expression can be used to distinguish effector T cells (T_E_) and effector memory T cells (T_EM_) from both naive T cells and central memory T cells (T_CM_) [28], [29]. Phenotypic analysis showed that upon comparing the percentages of the CD8^+^CD62L^+^ subset in TIL and matched PBL, a significant age-related decline (*P* = 0.019) was found (Fig. 5A). Conversely, there was no obvious age-related trend (*P* = 0.489) for the ratio of the CD45RO^+^CD8^+^ subset between matched TIL and PBL (Fig. 5B). In addition, no clear changes were observed in the CD8^+^CD62L^+^ subset (Fig. 5C), or the CD45RO^+^CD8^+^ subset of matched TIL and PBL (Fig. 5D). These data indicate that naive T cells and T_CM_, of the CD8^+^CD62L^+^ non-effector subset, significantly decreased in TIL with age, which correspondingly suggested a significant increase of T_E_ or T_EM_.

### Colocalization analysis of CD3 and CD8 molecules on TIL

The response of T cells to antigen stimulation is characterized by directed colocalization of CD8 molecules and TCR-CD3 complexes. To evaluate the immune status of TIL specimens that showed altered expression of specific TCR-Vβ subfamilies, colocalization of CD3 and CD8 on individual cells was detected by confocal laser scanning microscope (CLSM), after staining with fluorescent antibodies.

**Figure 6:**
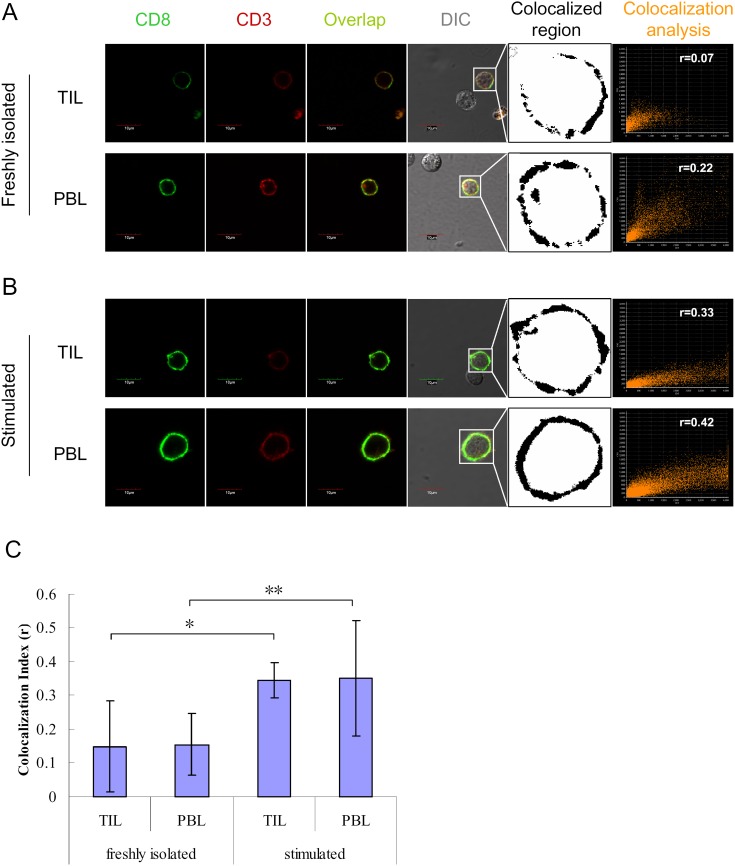
Colocalization analysis of CD3 and CD8 on TIL of patients and on PBL of healthy controls. TIL of cancer patients and PBL of healthy controls, either freshly isolated (**A**) or stimulated (**B**) with 1000 U/ml IFN-γ (at day 0), 50 ng/ml OKT3 (at day 1) and 500 U/ml IL-2 (from day 1 onward to day 6), were separately incubated with mAbs, CD8-FITC and CD3-PC5. The cells were observed with a confocal laser scanning microscope. The colocalization of CD3 and CD8 was analyzed, and colocalization index (r) was calculated by FV10-ASW2.1 software. For each group, 30 different cells from 3 individual were used for calculating the colocalization index (**C**). *P<0.05; **P<0.01, unpaired 2-tailed Student's *t* test.

A similar overlap of CD3 and CD8 molecules was observed on the surface of both freshly isolated TIL and PBL, and there was no evident difference between groups (Fig. 6A and C). After stimulation with cytokines and mAb, the colocalization of CD3 and CD8 was substantially enhanced both on TIL and PBL (Fig. 6B and C). These data suggest that both CD8^+^ TIL and PBL have comparable organization and mobilization of surface signaling molecules, and a similar functional status. The relatively weak colocalizaiton of CD3 and CD8 on freshly isolated TIL also suggests that effector TIL are functionally suppressed in tumor microenvironment.

## Discussion

The presence of TIL is thought to reflect the host's immune response to tumor cells and to be a valuable predictor of patient prognosis [5], [30]. A skewed TCR repertoire in TIL has been described in several studies [17]–[19], and in others, clonal expansion with restricted TCR repertoire was reported [16], [27]. However, many properties of TIL remain to be elucidated, including proliferation and clonality, variation of diversity, and the changing pattern of TCR subfamilies.

In the present study, both abnormally expressed TCR-Vβ subfamilies and a skewed TCR-Vβ repertoire were observed in TIL and PBL of cancer patients (Fig. 1). The degree and range of expression change of TCR subfamilies was different in different patients, which indicated that tumor induced immune responses vary widely in strength and breadth between individuals [30]. In addition, we found substantial expression differences of TCR-Vβ subfamilies between matched TIL and PBL of individual patients, suggesting that the infiltration of lymphocytes is not random and that antigen-driven clonal selection may occur in TIL [31], [32].

The Δ value was adopted to quantify the expression differences of TCR-Vβ subfamilies between TIL and PBL. Not surprisingly, Δ values differed in individual patients (Fig. 2A). To evaluate the dispersion degree of Δ values, the variance of multiple Δ values was calculated. It was found that, with highly statistical significance, older patients had the larger variance (Fig. 2B), mainly caused by changed expression of TCR-Vβ subfamilies in TIL, but not in PBL (Fig. 3). The increased variance of TCR-Vβ subfamily differences between TIL and PBL implies that a more limited number of highly activated TCR-Vβ subfamilies contribute to the immune response in TIL of older patients.

Using immunoscope analysis, we determined the clonal expansion pattern of TIL from lung cancer patients. A trend of decreasing diversity of TCR-Vβ repertoire with increasing age was apparent (Fig. 4). Although oligoclonal/monoclonal expansion increased with age, many TCR-Vβ subfamilies that were significantly overexpressed in TIL of older patients showed a polyclonal expansion pattern. These data indicated that most of the overexpressed TCR-Vβ subfamilies generally consisted of multiple responsive T cell clones.

It has been reported that the reduced diversity of the TCR repertoire in aged individuals may be caused by thymic involution, imbalanced T cell clonal expansion, or chronic latent viral infection [33]. Increased expression of some TCR subfamilies could also be induced by certain viral infections, such as cytomegalovirus (CMV), in aged patients. However, it is unlikely that viral infection induced the overexpression of TCR subfamilies observed in this study, considering that most of the TCR subfamilies showed significantly changed expression levels in TIL, but not in PBL. Therefore, as deduced above, the overexpressed TCR subfamilies in TIL may target the tumor cells themselves.

Thymic involution in older individuals is a key factor involved in TCR repertoire reduction [33]–[35]. T cell development takes place in the thymus, and continuous naive T cells that are exported (known as recent thymic emigrants or RTEs) maintain the diverse peripheral T cell repertoire [34]. It has been estimated that the complete naïve compartment can turn over in the course of 1–2 years [36]. However, the thymus progressively involutes with age. This process is thought to begin in humans as soon as 1 year after birth, when an initial reduction in thymic epithelial area can be observed [37]. With thymic involution, the diversity of the T cell repertoire is compromised, which results in impaired immune responses [38]. To compensate for the decline in naive T cells, memory T cells may participate in responses to unrelated antigens [34]. It was found that memory CD8 T cells could recognize cross-reactive epitopes from closely related or totally unrelated pathogens [39], [40]. Given the limited but intense change in TCR-Vβ subfamily expression found in the TIL of older patients (Fig. 2), it seems reasonable to surmise that memory cells are substantially involved in the tumor immune response.

The loss of surface CD62L expression can be used to distinguish T_E_ and T_EM_ from both naive T cells and T_CM_ [26], [28], [29]. Phenotypic analysis showed that CD8^+^CD62L^+^ subsets displayed no clear distribution trend in TIL and PBL from cancer patients when considered in the aggregate (Fig. 5C). However, when comparing the percentage of CD8^+^CD62L^+^ subsets in TIL to that in matched PBL from individual patients, it appears that a significant age-related decline takes place (Fig. 5A). These data indicated that the CD8^+^CD62L^+^ non-effector T cells subset (naive T cells and T_CM_) significantly decreased in TIL with age, which correspondingly suggested a significant increase of the CD8^+^CD62L^−^ effector T cells subset (T_E_ and T_EM_) [41]. Considering the reduced numbers of naïve T cells with age, the increased effector T cell subset may primarily consist of T_EM_ in older patients, which is in accordance with the more intensive expression change of some TCR-Vβ subfamilies (Fig. 1 and 2).

One of the phenotypic properties of effector T cells is the colocalization of CD8 and TCR-CD3 complexes. The TCR is excluded from lipid rafts in resting mature T cells. However, CD8 is a lipid raft-resident protein. Lipid rafts are important membrane microdomains for immune synapse formation, which serve to spatially segregate signaling components in the plasma membrane and regulate the initiation and prolongation of signaling [42]–[43]. The TCR-CD3 complex is recruited into lipid rafts at the initiation of T cell activation. Then CD3ζ chains are phosphorylated by the Src-family kinase, LCK, which is clustered by the CD8 cytoplasmic domain [44]. Therefore, colocalization of CD8 molecules and CD3 molecules is necessary for the activation of CD8^+^ T cells.

The colocalization of CD8 molecules and CD3 molecules on TIL was detected by CLSM (Fig. 6). These data showed that both CD8^+^ TIL from patients and PBL from healthy controls have comparable organization and mobilization of surface signaling molecules. The relatively weak colocalizaiton of CD3 and CD8 on freshly isolated TIL suggests that effector TIL are functionally suppressed in tumor microenvironment.

Considering the enhanced fluctuation of TCR subfamilies, decreased diversity of TCR repertoire, and increased effector phenotype, it is reasonable to infer that fewer clones showed more intense responses in the TIL of aged cancer patients. However, this property may be unfavorable for older individuals. It has been reported that some CTL clones lose their specific cytolytic activity and cytokine production under certain stimulation conditions, while retaining an activated phenotype and antigen-dependent growth pattern [45], [46]. This is akin to virus specific “Sisyphean” CD8 T cells found in persistently infected hosts [47]. Given the clinical tumor stages of subjects enrolled in this study, it does not appear that effector T cell proliferation led to obvious clinical improvement in aged individuals.

In conclusion, the age-related TCR repertoire variation and phenotypic alteration of memory T cells between TIL and PBL observed in this study provides insight into the development of effective immunotherapy for cancer patients of different ages.

## Supporting Information

Table S1 **Primers for amplification of TCRB CDR3 by multiplex PCR.**
(DOC)
