# Supplementary material for: Differences in TCR-Vβ Repertoire and Effector Phenotype between Tumor Infiltrating Lymphocytes and Peripheral Blood Lymphocytes Increase with Age
Source: PLoS One. 2014 Jul 14;9(7):e102327. doi: 10.1371/journal.pone.0102327 (PMC4096599; doi:10.1371/journal.pone.0102327)
Supplement: Table S1 — Primers for amplification of TCRB CDR3 by multiplex PCR. (DOC) [file pone.0102327.s001.doc]

**Supplementary File**

**Table 1**

Primers for amplification of TCRB CDR3 by multiplex PCR

| ***Primer type*** | ***Multiplex set*** | ***TCRBV^a^*** | ***Primer Sequences 5’ to 3’*** |
| --- | --- | --- | --- |
| Forward specific primers  (20nmol/L) | A | BV1 | AGGTGACACTATAGAATA CTTGCACTCTGAACTAAACC |
|  |  | BV2 | AGGTGACACTATAGAATA TACCGTTCCCTGGACTTTC |
|  |  | BV3 | AGGTGACACTATAGAATA CAAAGTAACCCAGAGCTCG |
|  |  | BV4 | AGGTGACACTATAGAATA CCTGGACAGAGCCTGACA |
|  |  | BV5 | AGGTGACACTATAGAATA GAGWVRVARAGGAAACTTCCCT |
|  |  |  |  |
|  | B | BV6 | AGGTGACACTATAGAATA RMKCTCAGGTGTGATCCAA |
|  |  | BV7a | AGGTGACACTATAGAATA AACCTTCACCTACACGCCC |
|  |  | BV7b | AGGTGACACTATAGAATA TBCCTTCACCTACACACCC |
|  |  | BV8 | AGGTGACACTATAGAATA ATGCRRGGACTGGAGTTG |
|  |  | BV9 | AGGTGACACTATAGAATA AATGAAACAGTTCCAAATCGC |
|  |  |  |  |
|  | C | BV11 | AGGTGACACTATAGAATA CGAGGAATGGAACTACACC |
|  |  | BV12a | AGGTGACACTATAGAATA TGAGATGTCACCAGACTGA |
|  |  | BV12b | AGGTGACACTATAGAATA TGACGTGTCACCAGACTTG |
|  |  | BV13 | AGGTGACACTATAGAATA ACTCAGACCCCAAAATTCC |
|  |  | BV13.5 | AGGTGACACTATAGAATA ATCACCCAGGCACCAACATCT |
|  |  | BV14 | AGGTGACACTATAGAATA ATAAGGGAGATGTTCCTGAA |
|  |  | BV15 | AGGTGACACTATAGAATA ATTCTCCCTGTCCCTAGAG |
|  |  |  |  |
|  | D | BV16 | AGGTGACACTATAGAATA TCAGTTCCCCAGCCACAG |
|  |  | BV17 | AGGTGACACTATAGAATA CAGAAAGGAGATATAGCTGAA |
|  |  | BV18 | AGGTGACACTATAGAATA GAGGAAGGTCTGAAATTCAT |
|  |  | BV20 | AGGTGACACTATAGAATA AGTTCATCCTGAGTTCTAAG |
|  |  |  |  |
|  | E | BV21 | AGGTGACACTATAGAATA CTCTCAAGATCCAGCCTG |
|  |  | BV22 | AGGTGACACTATAGAATA CCAGACTCCCAGCCATCA |
|  |  | BV23 | AGGTGACACTATAGAATA AAATGCTATCCTATCCCTAG |
|  |  | BV24 | AGGTGACACTATAGAATA CAATGAAGCAGACACCCCT |
| Reverse specific primer  (20nmol/L) | | TCRBCr | GTACGACTCACTATAGGGA CTCAAACACAGCGACCTC |
| Forward universal primer*  (800nmol/L) | |  | AGGTGACACTATAGAATA |
| Reverse universal primer  (800nmol/L) | |  | GTACGACTCACTATAGGGA |

The forward specific primers and reverse specific primer are separately fused with universal sequences (underlined) matched with universal primers; ^a^, Nomenclature from Arden et al; *, The primer is labeled by 5-Carboxyfluorescein.
